# The importance of soft skills development in a hard data world: learning from interviews with healthcare leaders

**DOI:** 10.1186/s12909-021-02567-1

**Published:** 2021-03-06

**Authors:** Traci H. Abraham, Greg L. Stewart, Samantha L. Solimeo

**Affiliations:** 1 VA Office of Patient Care Services, Primary Care Analytics Team-Iowa City, Iowa City VA Health Care System, Iowa City, IA USA; 2 Center for Mental Healthcare & Outcomes Research (CeMHOR), Central Arkansas Veterans Healthcare System, 2200 Fort Roots Rd., Bldg 58, North Little Rock, AR 72114 USA; 3 Department of Psychiatry, Center for Health Services Research (CHSR), University of Arkansas for Medical Sciences, Little Rock, AR USA; 4 Department of Management & Entrepreneurship, Tippie College of Business, University of Iowa, Iowa City, IA USA; 5 VA Office of Rural Health, Veterans Rural Health Resource Center- Iowa City, Iowa City VA Health Care System, Iowa City, IA USA; 6 Center for Access & Delivery Research & Evaluation (CADRE), Iowa City VA Health Care System, Iowa City, IA USA; 7 Department of General Internal Medicine, University of Iowa Carver College of Medicine, Iowa City, IA USA

**Keywords:** Healthcare leaders, Leadership training, Soft skills, Qualitative evaluation

## Abstract

**Background:**

Learning healthcare systems have invested heavily in training primary care staff to provide care using patient-centered medical home models, but less is known about how to effectively lead such teams to deliver high quality care. Research is needed to better understand which healthcare leadership skills are most utilized or in need of development through additional training.

**Method:**

Semi-structured telephone interviews with healthcare leaders familiar with Patient-Aligned Care Teams (PACT) implementation in the U.S. Department of Veterans Affairs (VA). We interviewed sixteen (*N* = 16) physician, nursing, and administrative leaders at VA facilities located in the upper Midwestern United States. Content analysis of interviews transcripts using template techniques.

**Results:**

Participants described instrumental challenges that they perceived hindered leadership effectiveness, including the supervisory structure; pace of change; complexity of the clinical data infrastructure; an over-reliance on technology for communication; and gaps in available leadership training. Factors perceived as facilitating effective leadership included training in soft skills, face-to-face communication, and opportunities for formal training and mentorship. A cross-cutting theme was the importance of developing “soft skills” for effective PACT leadership.

**Conclusions:**

Although formal leadership training and development were perceived as beneficial, healthcare leaders familiar with PACT implementation in the VA described a mismatch between the skills and knowledge PACT leaders need to succeed and the training available to them. Closing this gap could improve retention of skilled and knowledgeable healthcare leaders, thereby reducing the costs associated with training and leading to improvements in healthcare delivery.

## Background

Patient Aligned Care Teams (PACT), the Veterans Health Administration’s (VA) patient centered medical home model, has been implemented in more than 900 primary care clinics nationwide [1]. Successful medical home implementation has been associated with a reduction in use of specialty care, hospitalizations for ambulatory care sensitive conditions, and an increase in patient satisfaction with care [2–4] and thus a considerable amount of scholarship has sought to identify barriers to PACT implementation and function. Leadership support and skills have been identified as critical organizational resources to assure PACT implementation function. Because individual PACT members often report to different leaders within the clinic, PACT members rely upon local healthcare leadership to assist with resolution of within-team conflict resultion [5]. Notably, leadership support of the PACT model has been associated with: better recruitment and retention of PACT members [1]; stronger PACT teamwork [6]; greater sense of team member accountability to the PACT [5]; and better patient-reported access to care [7].

While some research has examined the role of primary care providers as de facto leaders of individual PACTs [8], less attention has been directed toward the needs, capacities, and role of healthcare leaders who oversee PACTs. Additionally, while many healthcare organizations provide leadership training, we know little about the types of training that are most effective or the specific skills that healthcare leaders need to develop to be effective [9]. This knowledge gap not only creates inefficiencies, but impedes the application of evidence-based principles to improve healthcare delivery [10, 11]. Thus, the objective of this study was to help close this gap by identifying healthcare leaders’ perspectives on the training and skills needed to effectively oversee and manage PACTs.

## Methods

### Overview

The research team consisted of two doctorally trained medical anthropologists (THA, SLS) who were health services researchers in the VA and an organizational psychologist (GLS). Our overall approach focused on obtaining descriptive data that could be used to inform clinical practice (i.e., identifying how to develop more effective healthcare leaders in clinical environments that have implemented PACT). To these ends, we analyzed interviews using template analysis, an approach derived from the tenants of content analysis with an emphasis on data synthesis and reduction, rather than interpretation [12]. Template domains were thus defined a priori by the goals of the study rather than a theoretical framework. This project was approved by the Institutional Review Board at the Iowa City VA Health Care System prior to data collection.

### Participants

Interviews were conducted by telephone with healthcare leaders located at VA facilities in the upper Midwestern United States. Sampling was purposive [13]. Regional executive leaders were asked to identify potential facility-level healthcare leaders to interview for the study. Twenty-four leaders with no prior relationship to the research team were invited to participate based in their role in the VA and knowledge of PACT implementation. Sixteen leaders (*N* = 16) agreed to an interview and none dropped out of the study. Recruitment occurred by e-mail and included an Information Sheet describing the project, project aims, and data collection procedures. Individuals in physician, nursing, and administrative leadership positions were sent an invitation to ensure that a diversity of experiences and perspectives were sampled.

### Data collection

Data were collected from April to May 2019 by THA using a semi-structured interview guide developed for this study to ensure consistency across interviews. Interview guide domains were informed by the goals of the study, and included: healthcare leader’s organization, role and perception of VA policies that create barriers to effective leadership; critical skills and knowledge of PACT leaders; resources that help healthcare leaders be effective; resources critical in participant’s own leadership role; and additional resource and training needs. A preliminary interview guide was piloted with initial participants; adjustments were made to questions and probes as needed. Interview guide available upon request. Interviews ranged from 30 to 60 min, were audio recorded with participants’ permission, and transcribed for analysis.

### Data analysis

Data from the interviews was analyzed from June to September 2019. The template contained the following a priori domains: role and responsibilities in organizational context; skills, knowledge, abilities and resources of effective leaders; and professional development. Inductive categories were developed by summarizing interview content within these a priori domains. To utilize each researcher’s analytic strengths, the team adopted a layered approach to templating the remaining interviews. THA developed an initial summary template for each interview, which was then audited for accuracy and completeness by SLS. Any questions about templated content or edits were resolved through discussion during team meetings. Although the aim of this study was to collect a broad range of experiences and perspectives, data collection was limited to leaders who volunteered to participate. We therefore cannot guarantee that saturation was reached. Data are available on request from the authors.

Rigor was established during the analytic process. A template rubric was developed by THA with domain names and definitions to control analytic drift. The process of independently templating the first two interviews permitted the identification of discrepancies in how domains and categories were interpreted by the two analysts. Finally, all templates developed by THA were audited for accuracy and completeness by SLS. To further ensure the accuracy of interpretation, primary results were disseminated to participants for feedback via report briefs.

## Results

### Participants

Sixteen (*N* = 16) VA healthcare leaders expressed their interest in participating by contacting the study coordinator to schedule a telephone interview. Table 1 summarizes participant characteristics. Participants described both challenges to effective PACT leadership and the resources and skills needed to overcome those challenges. In the following sections, we summarize lessons learned from interviews.

**Table 1:** Participant Clinical Role and Gender (*N* = 16)

Role	Male (***n*** = 5)	Female (***n*** = 11)	Total
Administrative Leaders	3	2	5
Nurse Leaders	0	7	7
Physician Leaders	2	2	4

### Barriers to effective PACT leadership

Participants described different instrumental challenges that made it difficult to be effective healthcare leaders both in the VA and in a PACT clinic environment. The primary challenges cited included: the pace of change within the VA; the supervisory structure for clinical personnel; complexity of the clinical data infrastructure; an over-reliance on technology-mediated communication, and gaps in available leadership training. Table 2 provides an overview of the primary challenges along with excerpts from the interviews to provide additional context, nuance or detail when needed.

**Table 2:** Barriers to Effective Healthcare Leadership: Categories, Subcategories, and Exemplars

BARRIERS	EXEMPLARS
**Supervisory structure**	
*Silo-ed priorities*	“The service line models are good. However, sometimes […] we kinda get stuck into our own way of doing things or own directives […] and it kinda does form pillars and work. Sometimes we’re not all on the same page” (P08).
*Lack of communication*	“Nursing doesn’t report at the service line level to the service line director and so you’ve got the silo of Nursing doing kinda whatever it wants and then the medical staff doing whatever they want and there’s no interaction” (P05).
**Pace of change**	“At the VA, we get things rolled down to us daily that need attention and action” (P10).
**Complexity of clinical data infrastructure**	
*Locating data*	“My biggest frustration is [that] I find a report I like and then for me to find that report again is impossible” (P05).
*Volume of data*	“I think the other struggle is that we have too much data and it’s all over the place” (P04).
**Over-reliance on technology for communication**	“I think a lot of work is done or sent through e-mail that really should be done with a face-to-face discussion or telephone meetings. A freer, easier exchange of ideas, really drilling down on processes and details, is what is really important in my job” (P15).
**Gaps in available leadership training**	
*Conflict management skills* *Skills at building cohesive teams*	“How do get people connected? I think that’s the biggest thing that we don’t train our leaders to do […] that storming and norming and conflict and all of that stuff” (P12).
*Motivating staff*	“We don’t have good manager trainings on how to use your soft skills a little more and how to motivate employees […] the intangible things” (P08).
*Delivering bad news*	“You don’t get a whole lotta training on how [to] be the bad guy. The crucial conversations training. I think that should be required for every leader” (P04).
*Formal mentorship program*	“You should create a mentorship program where you pair up people that have done this for periods of time in their life, or are considered to be extremely effective in this area, and partner them up with people that are just starting out” (P10).

One significant challenge was the organization of team members into silo-ed supervisory structures, or service lines (e.g., Primary Care, Nursing). This structure made it difficult to develop and advance shared priorities within the PACT and hindered communication. Organizing services within independent service lines sometimes led to “silo-ing” (i.e., a communication breakdown). Silo-ing furthermore superseded other, more tangible barriers to effective leadership. In this respect, one participant stated: “When you’re siloed, it doesn’t come down to resources or time or any of these other things. If all the members of the PACT team have different supervisors, it’s really hard for the PACT leader if those supervisors are not on the same page” (P11).

Another challenge was the pace of change within a learning healthcare system dedicated to providing evidence-based healthcare. The pace of change was seen to outpace development of implementation guidance for new clinical policies, processes or practices. For example, one participant observed with trepidation: “The [new program] is gonna be one of them where there’s gonna be huge changes, and we don’t have clear guidance about what we need to be working on” (P08).

Data infrastructure also hindered effective leadership, including both the fragmentation and volume of data available to leaders at the VA. Some participants described challenges locating the data they needed to make key decisions stemming from a fragmented data infrastructure. These challenges were compounded by the sheer volume of data available to leaders.

An additional barrier was a perceived over-reliance on technology in place of face-to-face communication. Technologically medicated communication was perceived by participants as limited “open” communication. Some participants also expressed that it increased the likelihood of miscommunication and thus errors.

A final challenge observed by leaders was gaps in the training available to them that hindered the development of effective PACT leaders. Participants most often cited a need for additional training in interpersonal, or “soft skills,” such as conflict management, teambuilding, motivating staff, and delivering bad news. Participants noted that physician leaders, in particular, often required supplemental training to help them better communicate with direct reports. Participants also recommended developing a more formal mentorship program for healthcare leaders. Additional gaps described less often included instruction in how to complete administrative tasks, including managing contracts, completing payroll forms, and personnel actions (e.g., performance appraisals, disciplinary procedures) and guidance in how to connect the tools at their disposal to daily practice, especially in respect to informatics training.

### Facilitators of effective PACT leadership

Participants also described facilitators which made healthcare leaders more effective. Current resources and skills identified as facilitating effective leadership, such as training programs and informal mentoring, highlighted existing supports used in overcoming leadership challenges. Participants also emphasized the importance of soft skills and face-to-face communication in the ability of PACT leaders to be effective. Table 3 provides an overview of the primary facilitators along with exemplars from the interviews which provide contextual, nuanced detail.

**Table 3:** Facilitators to Effective Healthcare Leadership: Categories, Subcategories, and Exemplars

FACILITATORS	EXEMPLARS
**Soft skills**	
*Communication skills*	“Our job is to coordinate, communicate, and collaborate with all the folks trying to provide care out in the clinics. So, if you don’t have that ability to communicate, you’re gonna be very ineffective in this position” (P03).
*Responding positively to adversity and stress*	“I think that adaptability and organization are probably the two most critical [skills] in the VA, because things change all of the time. So, you have to be able to adapt at a moment’s notice to a change of practice, a change of procedure” (P01).
*Conflict management skills*	“It’s conflict management, conflict resolution when you have PACT--, parts of PACT teams that may not function the way they need to. You know, there’s, whether it’s a personality conflict or something else” (P14).
*Listening skills*	“There’s a lot of emails that fly back and forth, there’s a lot of phone calls, so being willing to listen constantly, you will probably be able to pick up on things you need” (P16).
**Face-to-face interaction**	“We have a what we call a hallway meeting every Tuesday at 11:00. It would be extremely inefficient if we didn’t actually meet to communicate with all the people that need to know the information or need to contribute to make decisions” (P11).
*Builds PACT cohesion*	“I’m like: ‘You know, since we’re not quite the cohesive group that we maybe once were, we need to build relationships,’ and now we have very frequent [face-to-face] meetings” (P08).
*Provides networking and mentoring opportunities*	“We do have [in-person] meetings that happen twice a year. I think that that’s been helpful to kind of network with those leaders, talk with them, be around them, pick up on what they’re doing” (P15).
**Informal mentorship**	“Most of my leadership skills have been acquired through mentorship through people who I have worked with who really are good and smart and dedicated” (P15).

Leaders described soft skills as particularly important for PACT leaders. Primary among soft skills was an ability to communicate, which helped PACT leaders coordinate clinic staff and create a common culture or mission amongst PACT team members. One participant explained: “You’re trying to get a lot of disciplines that aren’t inter-related to work together towards the common goal of taking care of patients. Nurses approach things differently than techs, than docs, than administrators. So, first and foremost, is communication and being a good conductor or a team builder” (P03). Participants also described soft skills related to mediation and conflict management as critical for PACT leaders. Responding positively and/or proactively to adversity and stress, referred to as “adaptability,” “resilience” or “flexibility,” was also cited as an important soft skill which helped healthcare leaders to overcome the challenges cited above. For example, one participant responded when asked to describe skills possessed by effective leaders: “Flexibility, ‘cause there are lots of changes that go through PACT and the ability to adapt is very much needed” (P05). A final soft skill of effective healthcare leaders was an ability to listen, which were described as important for overcoming challenges posed by an overreliance on technology for communication.

Leaders also cited face-to-face interactions as facilitating effective PACT leadership. In-person meetings were often described as vital to day-to-day decision-making and furthermore helped leaders build a cohesive PACT clinic culture. Face-to-face interactions also provided opportunities to interact with peers and develop a support network that could be drawn upon to fill gaps in formal leadership training.

Leaders also described formal training programs as having helped them acquire some of the skills and knowledge they needed to be effective, particularly in a PACT clinic environment. Amongst the trainings deemed helpful was a comprehensive national leadership effectiveness, accountability, and development program, and specific training related to team development and facilitation. Local training initiatives were also cited as effective, including an informal shadowing program for mid-level clinic managers focused on helping new leaders form a “buddy system” with more experienced healthcare leaders. A final facilitator was informal mentoring. In this respect, both opportunities for ongoing mentoring and having an ability to travel to another clinic and receive hands-on mentoring were cited as helpful.

## Discussion

During telephone interviews, VA healthcare leaders described the instrumental challenges involved in leading within an organizational environment of rapid change and a complex, potentially silo-ed supervisory structure in which a fragmented and deep data infrastructure made it difficult to make informed decisions. These challenges were compounded by gaps in training and an over-reliance on technology in place of face-to-face interaction. The importance of interpersonal, or “soft skills,” in overcoming the challenges they encountered in their everyday practice emerged as a cross-domain theme. Analysis thus identified a disconnect between the challenges faced by healthcare leaders, both in PACT and in general, the expertise they typically associated with effective leadership, and the training available to leaders.

Emerging scholarship examining the impact of effective healthcare leaders suggests that physician leaders may produce better quality care than non-clinically trained healthcare executives [14]. There is, however, a paucity of evidence demonstrating a relationship between specific leadership skills and clinical outcomes. Lacking an evidence base to guide investment in specific leadership training foci, our study’s findings, derived from interviews with senior healthcare leaders, signal a need to develop interpersonal skills in healthcare leaders both in PACT clinical environments and in general to help them be more effective at overcoming the challenges they encounter in their practice.

Participants reported that a significant barrier to effective healthcare leadership was gaps in the formal training available to them. As a result of these gaps, leaders relied upon their relationships with colleagues and informal mentoring to obtain knowledge and skills. Participants also noted a mismatch between their formal training and the skills needed to be effective in their everyday practice. Their experiences are not unusual, as other research has demonstrated that clinician’s leadership training is infrequent and relies heavily on peer mentoring [14, 15], with formal training prioritizing managerial rather than leadership skills [16, 17].

Although healthcare leaders in this study reported a need for managerial skills development, consistent with other research, they emphasized the importance of leadership or “soft skills” in their everyday practice [18]. ,including leading interdisciplinary PACT teams [19]. Participants described how communication, conflict resolution, listening, and collaborative skills helped them to overcome some of the challenges they encountered as leaders, such as an over-reliance on technology for communication. They also reported having used interpersonal skills to build relationships with colleagues, overcome the limitations of their managerial skills, and navigate within inflexible organizational structures (i.e., silo-ed service lines). Finally, participants observed that soft skills were fundamental to creating a common culture and sense of mission among PACT clinicians from different disciplines that is fundamental to delivering high quality healthcare.

The interview data we present here thus lend support to the growing attention to developing physician leader capacity for communication and collaboration [15, 17, 20]. The need to more purposively develop leaders’ interpersonal skills will only become more urgent as the use of technology to facilitate communication occurs ever more frequently. The emerging consensus that soft skills are fundamental for effective healthcare leadership is concomitant with the surfacing of research gaps pertaining to the impact of those skills on clinical or other outcomes [18]. Results from this study thus signal a need to close the knowledge gap between healthcare leader skills and clinical or other outcomes to support the development of evidence-based principles to improving healthcare.

Our findings suggest that closing the gap between the skills needed to be effective and available formal training could improve retention of skilled healthcare leaders, thereby reducing the costs associated with training. Evidence suggests that effective healthcare leaders furthermore decrease burnout and increase job satisfaction among physicians whom they oversee [21]. As participants both explicitly and implicitly referenced the importance of soft skills for being effective in clinical environments in which PACT has been implemented, we assert that providing leaders with training in soft skills could also enhance PACT functioning and thereby improve healthcare delivery. These findings align with the principles of complexity leadership, which highlights leadership as a complex relational process which occurs between people within an organization [22].

## Strengths and limitations

This small study allowed VA healthcare leaders to describe, in their own words, factors which they perceived as affecting their effectiveness. Findings from this exploratory study provide preliminary data which can be used to inform the development of larger studies, both within the VA and in private sector healthcare systems, leading to more generalizable findings. Our findings are limited in that they result from interviews with healthcare leaders in clinics located in a specific geographical area of the U.S. (i.e., the Upper Midwest). Additionally, analytic results reflect the experiences and perspectives of healthcare leaders in the VA, which is structured and organized differently from other healthcare systems in the U.S. and elsewhere.

## Conclusion

Although learning healthcare systems, such as the VA, have invested heavily in training primary care staff, gaps remain in our knowledge of which leadership skills are most utilized or in need of development. To begin to fill this gap, we interviewed sixteen (*N* = 16) VA physician, nursing, and administrative leaders. Healthcare leaders described instrumental challenges which they perceived hindered their effectiveness (e.g., the VA supervisory structure), as well as factors which they perceived as facilitating effective leadership (e.g., soft skills training). In general, VA healthcare leaders described a mismatch between the skills and knowledge they needed to succeed and the training available to them. Closing this gap could improve retention of skilled and knowledgeable healthcare leaders, reduce the costs associated with training, and lead to improvements in healthcare delivery.

## Data Availability

The datasets generated and/or analyzed during the current project are publicly available upon written request to the lead author.

## Abbreviations

PACT: Patient-Aligned Care Teams
VA: U.S. Department of Veterans Affairs
